# Tetralogy of Fallot and atrial septal defect in a white Bengal Tiger cub (*Panthera tigris tigris*)

**DOI:** 10.1186/1751-0147-56-12

**Published:** 2014-03-04

**Authors:** Paolo Pazzi, Chee K Lim, Johan Steyl

**Affiliations:** 1 Department of Companion Animal Clinical Studies, Faculty of Veterinary Science, University of Pretoria, Private Bag X04, Onderstepoort 0110, South Africa; 2 Diagnostic Imaging Section, Department of Companion Animal Clinical Studies, Faculty of Veterinary Science, University of Pretoria, Private Bag X04, Onderstepoort 0110, South Africa; 3 Current address: Department of Veterinary Clinical Sciences, College of Veterinary Medicine, Purdue University, West Lafayette, IN 47907-2026, USA; 4 Section of Pathology, Department of Paraclinical Sciences, Faculty of Veterinary Science, University of Pretoria, Private Bag X04, Onderstepoort 0110, South Africa

**Keywords:** Pentalogy, Pulmonic stenosis, Echocardiography, Necropsy

## Abstract

A 3-week-old female white Bengal Tiger cub (*Panthera tigris tigris*) presented with acute onset tachypnoea, cyanosis and hypothermia. The cub was severely hypoxaemic with a mixed acid–base disturbance. Echocardiography revealed severe pulmonic stenosis, right ventricular hypertrophy, high membranous ventricular septal defect and an overriding aorta. Additionally, an atrial septal defect was found on necropsy, resulting in the final diagnosis of Tetralogy of Fallot with an atrial septal defect (a subclass of Pentalogy of Fallot). This report is the first to encompass arterial blood gas analysis, thoracic radiographs, echocardiography and necropsy findings in a white Bengal Tiger cub diagnosed with Tetralogy of Fallot with an atrial septal defect.

## Background

Tetralogy of Fallot (TOF) is a rare and complex congenital cardiac disorder characterised by ventricular septal defect (VSD), right ventricular outflow tract narrowing or obstruction (pulmonic stenosis [PS]), overriding aorta and secondary hypertrophy of the right ventricle. TOF has been reported in dogs [1,2], cats [3-5], horses [6], cattle [7], sheep [8], an European beaver [9], a Japanese macaque [10], and an European brown bear [11]. The incidence of TOF in dogs diagnosed with congenital heart disease is approximately 0.6-1% [1,2] and the condition is considered even rarer in the cat with only a few case reports documented to date [3-5]. Pentalogy of Fallot (POF) is a rare variant of the relatively more common TOF, comprising the aforementioned four classic features of TOF with an additional atrial septal defect (ASD) or patent ductus arteriosus (PDA). POF has previously been described in three dogs [12-14], two horses [15,16], a ram [17] and as a necropsy finding in a two-year-old Siberian Tiger [18], however, of these reports, only a Korean Sapsaree dog [13] and the Siberian Tiger [17] have been diagnosed exclusively with TOF and an ASD.

The haemodynamics of TOF depends largely on the degree of right ventricular outflow tract obstruction. The VSD is usually nonrestrictive and if right ventricular outflow obstruction is severe, the intracardiac shunt is from right to left and pulmonary blood flow may be markedly diminished with deoxygenated blood being pumped into circulation resulting in cyanosis. The right ventricular hypertrophy is secondary to the pressure overload created by the PS and impingement of the interventricular septum on the right ventricular outflow tract, rather than a primary embryological malformation.

This is the first description of TOF with ASD (a subclass of POF) to include arterial blood gas analysis, diagnostic imaging and necropsy findings in a white Bengal Tiger (*Panthera tigris tigris*).

## Case presentation

A 3-week-old female white Bengal Tiger cub presented with a history of one day anorexia and tachypnoea. The cub suckled from the mother for one week, and was bottle fed thereafter. The cub was stunted and approximately half the size of her litter mates. On clinical examination the cub was in severe respiratory distress with increased expiratory effort, increased lung sounds with severe cyanosis of the mucous membranes. Although tachycardia was present (180 beats/minute), a murmur could not be detected most likely due to the expiratory lung noises. Mild hypothermia (36.9°C, normal range: 38.0-39.0°C) was also present. Initial management included oxygen supplementation and 0.1 mg/kg of butorphanol (V-Tech Pharmacy, Midrand, South Africa) intramuscularly, subsequently reducing the patient’s respiratory distress and reducing the severity of cyanosis. Serum biochemistry and electrolytes revealed no significant abnormalities, arterial blood gas showed severe hypoxia - partial arterial pressure of oxygen: 27.1 (normal range: 75–100 mmHg), mild acidosis pH: 7.341 (normal range: 7.350-7.450), low bicarbonate 13.2 (normal range: 20–24 mmol/L) and low partial arterial pressure of carbon dioxide (p_a_CO_2_): 20.9 (normal range: 32.0-45.0 mmHg).

**Figure 1:**
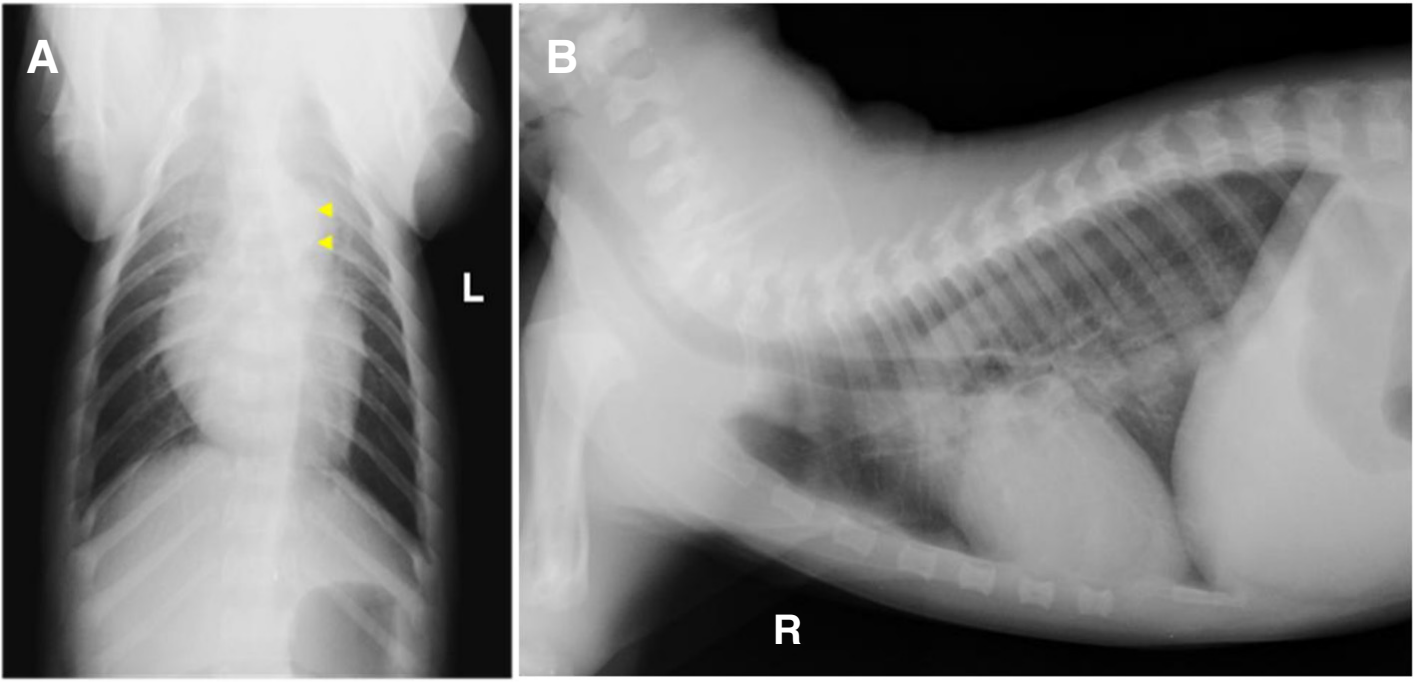
**Dorsoventral (A) and right lateral (B) thoracic radiographs.** Prominent main pulmonary artery bulge seen superimposing over the descending aorta on dorsoventral view and corresponding to soft tissue opacity at the cranial aspect of the heart base on the orthogonal view. Arrow heads point at the bulge in the pulmonary artery.

A prominent main pulmonary arterial bulge was seen superimposing over the aorta on the dorsoventral thoracic radiograph, corresponding with a soft tissue bulge at the cranial aspect of the base of the cardiac silhouette on the right lateral thoracic radiograph (Figure 1). There was a mild but inconsistent increased interstitial lung pattern seen in the cranial cupula and ventral aspect of the caudal lung lobes (seen only on lateral but not on dorsoventral view). The overall radiological findings were suggestive of pulmonic stenosis with post-stenotic main pulmonary artery dilatation and therefore, echocardiography was subsequently performed.

**Figure 2:**
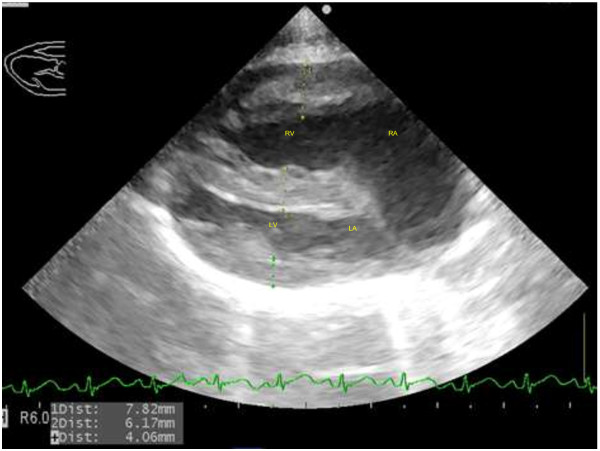
**Right parasternal long axis view of the heart with severe right atrial enlargement and moderate right ventricular hypertrophy.** Note the thickening of the right ventricular free wall and interventricular septum. Right atrium (RA), right ventricle (RV), left atrium (LA), left ventricle (LV).

**Figure 3:**
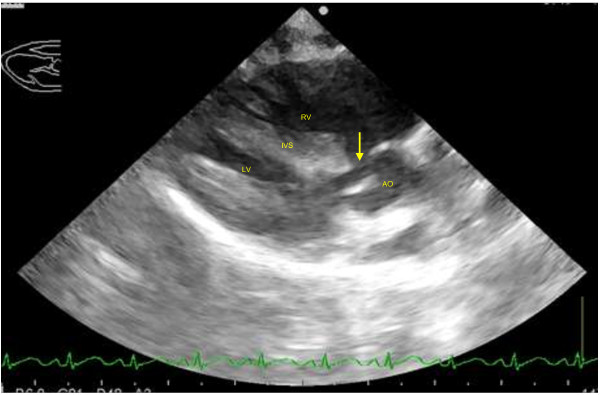
**Right parasternal long axis left ventricular outflow tract view of the heart with ventricular septal defect and overriding aorta.** A high membranous ventricular septal defect (arrow) is visible at the subaortic region. Right atrium (RA), right ventricle (RV), left atrium (LA), left ventricle (LV), interventricular septum (IVS).

**Figure 4:**
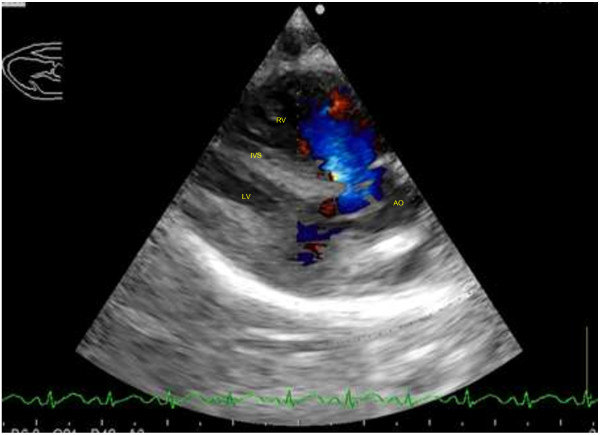
**Right parasternal long axis left ventricular outflow tract view of the heart with colour flow Doppler with a reversed ventricular septal defect shunt.** A right to left shunt characterised by blue-colour flow across the high membranous ventricular septal defect with concomitant overriding aorta. Right atrium (RA), right ventricle (RV), left atrium (LA), left ventricle (LV), interventricular septum (IVS).

**Figure 5:**
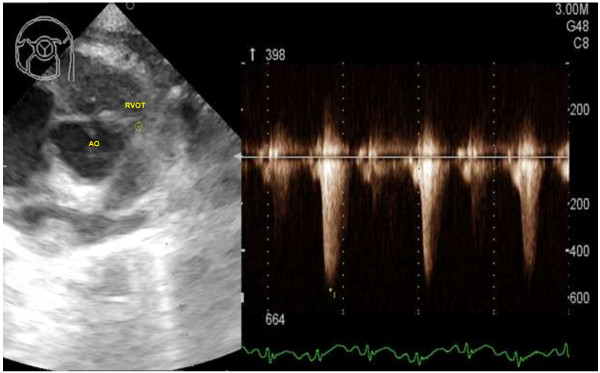
**Right parasternal short axis view of the heart base with right ventricular outflow tract and continuous wave Doppler showing severe subvalvular pulmonic stenosis.** There is marked narrowing of the right ventricular outflow tract with post-stenotic peak velocity of 564.5 cm/s. Right ventricular outflow tract (RVOT), aorta (AO).

On the right parasternal long axis echocardiography view, moderate thickening of the right ventricular free wall (two times the thickness of the left ventricular free wall) and interventricular septum (1.5 times the thickness of the left ventricular free wall) was visible, indicating moderate right ventricular hypertrophy (Figure 2). Marked enlargement of the right atrium was noted with severe, turbulent high velocity trans-tricuspid regurgitation up to 559 cm/s detected on continuous wave Doppler. On investigation of the left ventricular outflow tract, a high membranous ventricular septal defect (up to 2.4 mm wide) with concomitant overriding aorta was appreciable (Figure 3). A right-to-left ventricular shunt was detected on colour flow Doppler (Figure 4) with peak velocity up to 185 cm/s on spectral Doppler, with the majority of the shunted blood directed towards the left ventricular outflow tract and subaortic region. On the right parasternal short axis view, severe subvalvular PS characterised by marked narrowing of the right ventricular outflow tract was seen with post-stenotic peak velocity of 565 cm/s (Figure 5). Severe patient tachypnoea during the echocardiographic examination resulted in marked cardiac excursion and made it impossible to obtain an accurate M-mode tracing. Nevertheless, with the overall findings of high membranous VSD, overriding aorta, severe PS and right ventricular hypertrophy, a tentative diagnosis of TOF with a right-to-left VSD shunt was made.

**Figure 6:**
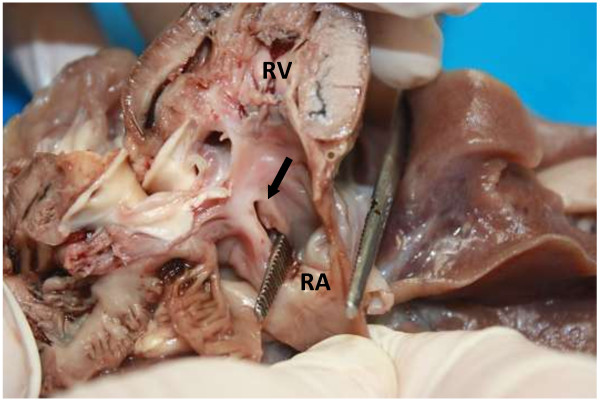
**Sagittal section through the right ventricle (RV).** There is an atrial septal defect (ostium secundum) between the right (RA) and left atrium (arrow and forceps). The right atrium is also significantly dilated.

**Figure 7:**
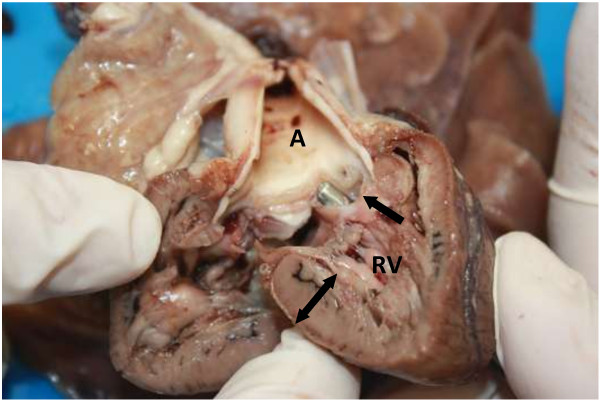
**Sagittal section through the right ventricle (RV).** An overriding aorta (A) communicating with the right ventricle (RV) and left ventricle through a subaortic ventricular septal defect (arrow and forceps). The right ventricular wall showed marked hypertrophy (double arrow).

**Figure 8:**
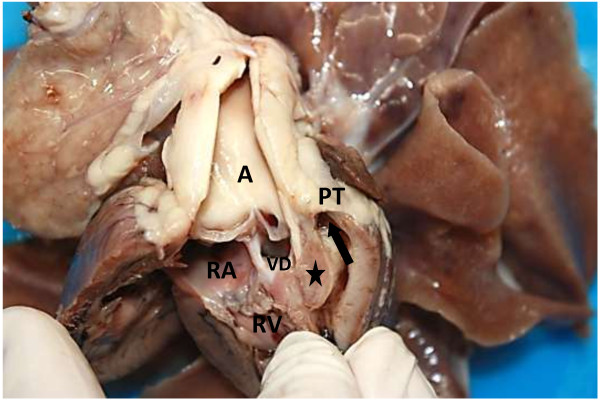
**Sagittal section through the right ventricle (RV).** A significantly hypoplastic pulmonary trunk (PT) demonstrates markedly reduced pulmonary arterial blood flow (arrow). Note the compressive effect (stenosis) of a hypertrophic proximal interventricular septum (star) on the pulmonary trunk (PT). Overriding aorta (A). Ventricular septal defect (VD). Right atrium (RA).

**Figure 9:**
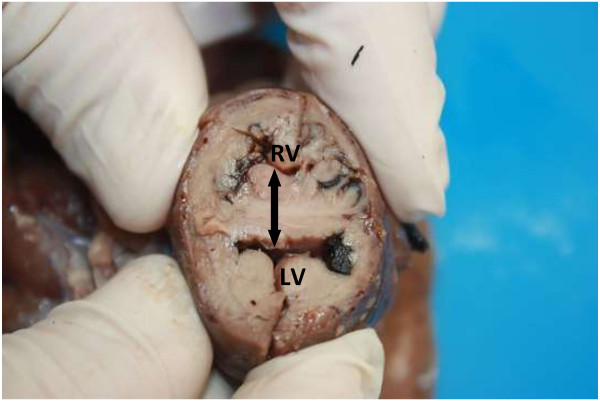
**Cross section through the mid right & left ventricular region (LV).** The right ventricle (RV) shows severe concentric hypertrophy due to pulmonary arterial stenosis. Note the marked ventricular septal hypertrophy (double arrow).

**Figure 10:**
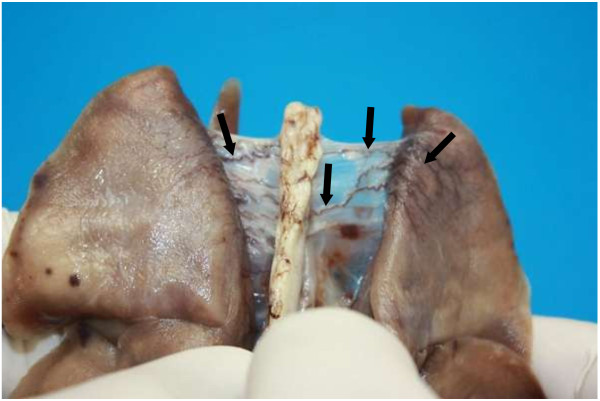
**Dorsal view of the lungs and bisecting thoracic aorta.** There are numerous prominent small tortuous thin walled blood filled vessels (veins) between the caudal pulmonary pleurae and periaortic mediastinal connective tissue (arrows). They originate as fine hair-like vessels in the pulmonary pleura and anastomose to form larger vessels towards the aortic adventitia where they drain into the azygos and costal veins (not in picture).

Due to the severity of the condition and the poor prognosis the patient was euthanased and a necropsy conducted. The multiple echocardiographic findings were confirmed during macroscopic examination and included an overriding aorta associated with a subaortic VSD, concentric right ventricular hypertrophy and right atrial dilatation, subpulmonary stenosis associated with localised ventricular septal hypertrophy resulting in pulmonary valve and trunk hypoplasia and aortic trunk dilatation. In addition, an ASD, consistent with an ostium secundum was found (Figures 6, 7, 8, and 9). The caudal thoracic periaortic mediastinum exhibited multiple prominent small tortuous blood filled vessels (veins) extending between the azygos - and costal veins and dorsocaudal pulmonary pleura (Figure 10). Prominent coronary veins due to marked venous dilatation could also be detected macroscopically.

No significant histopathological changes on routine haematoxylin & eosin staining could be demonstrated in myocardial fibres. Marked coronary vein dilatation histologically supported the macroscopic observation. The lungs showed generalised alveolar micro-atelectasis associated with pulmonary arterial collapse and hypoplasia due to poor pulmonary arterial perfusion. The bronchial and terminal bronchiolar veins were generally significantly distended. Histologically, the caudodorsal pulmonary pleural findings supported the macroscopic observation of prominent venous dilatation in pleural adventitia. Of other organs examined histologically, only the liver showed significant change, manifesting as moderate global hepatic venous dilatation.

## Discussion

Tetralogy of Fallot results from abnormal embryonic development of the conotruncal septum, resulting in varying degrees of infundibular and valvular PS, pulmonary artery hypoplasia, malalignment of the infundibular septum, and a VSD [19]. Specific genetic associations with TOF in humans include alterations in *JAG1*[20], *NKX2-5*[21], *ZFPM2*[22] and VEGF [23] while in dogs the inbreeding of Keeshond dogs led to the suspicion of a polygenetic threshold inheritance model for TOF [24,25]. Specific genetic associations have not been elucidated in dogs. Concurrent developmental abnormalities in addition to those causing TOF lead to ASD or PDA and resultant POF. TOF with ASD is a very rare condition and has been reported in only 2 animal species as sporadic individual case reports [13,17]. The white colour of Bengal Tigers is due to a recessive trait with selective inbreeding in captivity often encouraging the expression of recessive traits, and although TOF has not been associated with Bengal or white Tigers to date, abnormalities of the visual pathways have been associated with white Tigers [26]. The clinical presentation of the cub with cyanosis and tachypnoea was supported by the arterial blood gas that demonstrated severe hypoxaemia and concurrent mixed acid–base disturbance (metabolic acidosis and respiratory alkalosis) due to CO_2_ partial pressure lower than would be expected for pure compensation for the metabolic acidosis. The metabolic acidosis was most likely secondary to anaerobic cellular metabolism due to severe hypoxia, resulting in lactate accumulation. The lower-than-expected p_a_CO_2_ (considering the degree of peripheral cyanosis) was most likely a result of the severe tachypnoea due to the hypoxaemia, causing CO_2_ to be “blown-off” as well as only a mild right-to-left shunt seen on Doppler echocardiography. A larger shunt fraction/pressure may have resulted in greater p_a_CO_2_.

The radiological findings were supportive of PS with post-stenotic dilatation of the pulmonary artery but the pulmonary pattern was not typical for cardiogenic pulmonary oedema. In contrast to previous reports in dogs [12-14], diffuse cardiomegaly was not visualised in this Tiger. This may be due to the fact that the right-to-left ventricular septal defect shunting blood was directed into the subaortic region, thus minimising the effect of volume overload of the left heart while the moderate concentric hypertrophy of the right ventricle was not appreciable on radiographs.

The echocardiographic findings in this case were typical for a TOF and surprisingly the ASD was only detected during necropsy. Failure to identify the ASD on echocardiography was most likely due to the small size of the ASD while the absence of obvious shunting between the two chambers on colour flow Doppler was likely due to the equalisation of pressures between atria. The pulmonary outflow pressure in this cub was mildly increased compared to the previously reported value in the Korean Sapsaree dog also diagnosed with TOF and ASD [13]. The ventricular right-to-left shunt velocity measured in the Tiger cub may have included the left ventricular outflow tract due to the concomitant overriding aorta and could have resulted in a measured shunt velocity that is not a true reflection. Interestingly the right-to-left shunt velocity was of lower velocity than reported for the Korean Sapsaree dog [13], possibly related to the size of the VSD’s. The authors recommend if the classic findings of a TOF are diagnosed, it is advised to thoroughly exclude the possibility of a PDA or ASD to ensure the diagnosis of a POF is not missed.

The necropsy findings were very similar to the previously described adult Siberian Tiger [18], except no endocardiosis of the mitral valve was present in this Tiger cub. The other significant difference was the presence of locally extensive pleural venous distension in the caudal thoracic peri-aortic mediastinum covering the area between the azygos vein and dorsocaudal pulmonary pleura in this cub. The PS resulted in progressive right ventricular hypertrophy which increased the degree of pulmonary truncal stenosis, resulting in diminishing pulmonary arterial pressure. Diminished pulmonary arterial pressure explains the pathological findings of pulmonary arteriolar collapse and hypoplasia associated with suspected increased flow resistance to the bronchial arterial supply of the lung. This would result in most of the bronchial arterial supply being shunted to the bronchial venous system (normally most of the bronchiolar arterial supply drains into the pulmonary arterial flow via anastomosis), causing distension of bronchial and pleural veins draining into the azygos and costal venous system. Coronary vein distension was most likely as a result of increased right atrial pressure subsequent to tricuspid valve insufficiency secondary to PS.

## Conclusions

This report documented the first clinical case of TOF with ASD (a subclass of POF) in a Bengal Tiger with clinical and arterial blood gas signs of hypoxaemia, radiological and echocardiographic evidence of TOF and necropsy findings consistent with TOF with ASD. The reported findings may assist in the antemortem diagnosis of Tetralogy or Pentalogy of Fallot in other species.

## Abbreviations

ASD: Atrial septal defect; paC02: Arterial partial pressure of carbon dioxide; pa02: Arterial partial pressure of oxygen; PDA: Patent ductus arteriosis; POF: Pentalogy of Fallot; PS: Pulmonic stenosis; TOF: Tetralogy of Fallot; VSD: Ventricular septal defect.

## Competing interests

The authors declare that they have no competing interests.

## Authors’ contributions

PP was the primary clinician on the case, collated all clinical and imaging information and is the primary author of the paper. CKL carried out the diagnostic imaging procedures and interpretation. JS performed the necropsy and histopathology examination and interpretation. All authors made intellectual contributions, reviewed and approved the final manuscript.
